# Supplementary material for: Selection and validation of internal control genes for quantitative real-time RT‒qPCR normalization of Phlebopus portentosus gene expression under different conditions
Source: PLoS One. 2023 Sep 27;18(9):e0288982. doi: 10.1371/journal.pone.0288982 (PMC10530043; doi:10.1371/journal.pone.0288982)
Supplement: S1 Raw images — 0–12 lanes: CK, MSF1, SPRY, EF2, RAN, EIF, UBCE, EF1, MAPK, TBP1, SYB, Actin, CYP. X: marker. (PDF) [file pone.0288982.s002.pdf]

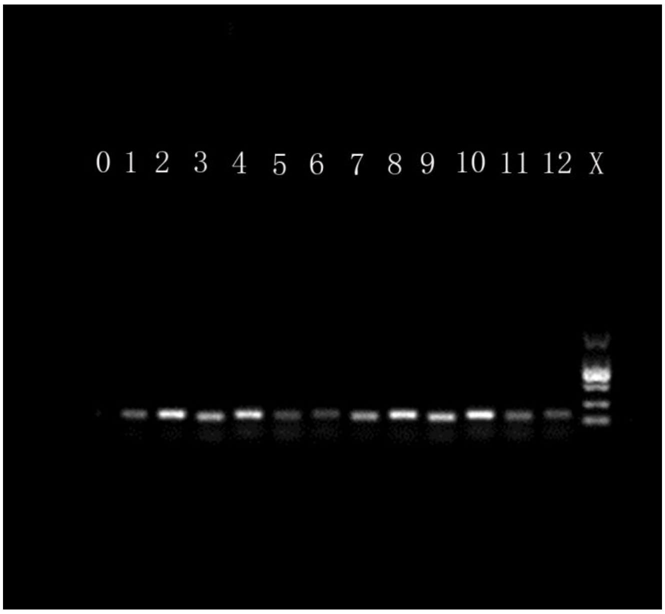

S1 Fig. The Amplified fragments of candidate reference genes shown by agarose gel electrophoresis. 0-12 lanes: CK, *MSF1, SPRY, EF2, RAN, EIF, UBCE, EF1, MAPK, TBP1, SYB, Actin, CYP*. X: marker.
