# Supplementary figures and images for: Selection and validation of internal control genes for quantitative real-time RT‒qPCR normalization of Phlebopus portentosus gene expression under different conditions
Source: PLoS One. 2023 Sep 27;18(9):e0288982. doi: 10.1371/journal.pone.0288982 (PMC10530043; doi:10.1371/journal.pone.0288982)

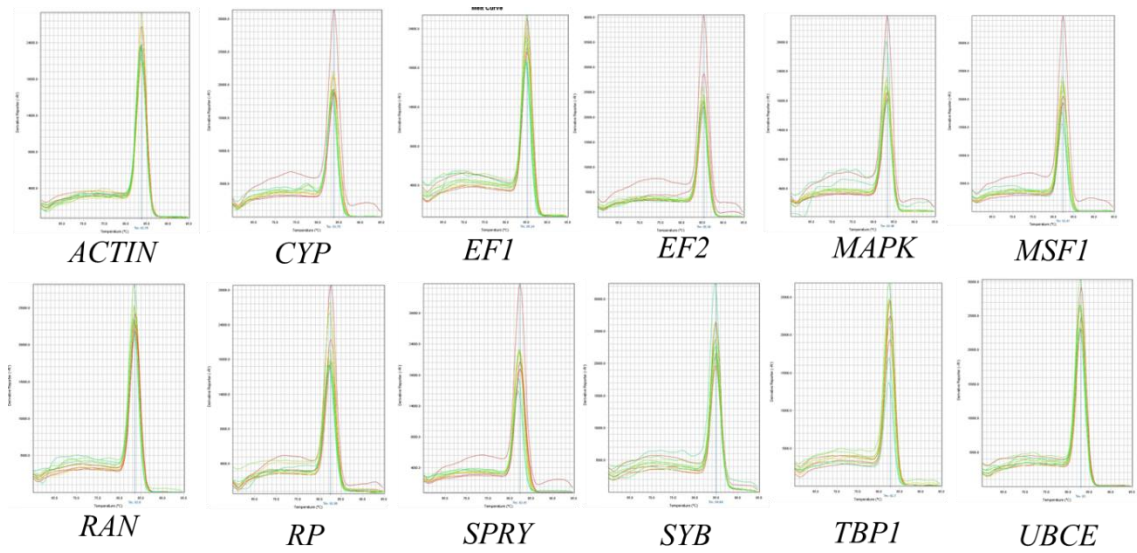

**S2 Fig.** Melting curve generated by qRT-PCR for 12 reference genes

Supplement: S1 Fig — (PDF) [file pone.0288982.s003.pdf]
